# Comparison between influenza coded primary care consultations and national influenza incidence obtained by the General Practitioners Sentinel Network in Portugal from 2012 to 2017

**DOI:** 10.1371/journal.pone.0192681

**Published:** 2018-02-13

**Authors:** Rosália Páscoa, Ana Paula Rodrigues, Susana Silva, Baltazar Nunes, Carlos Martins

**Affiliations:** 1 Family Medicine, Department of Community Medicine, Information and Health Decision Science, Faculty of Medicine of the University of Porto, Porto, Portugal; 2 Epidemiology Department, National Institute of Health Doutor Ricardo Jorge, Lisboa, Portugal; 3 Public Health Research Centre, Universidade Nova de Lisboa, Lisboa, Portugal; 4 CINTESIS—Centre for Health Technology and Services Research, University of Porto, Porto, Portugal; Columbia University, UNITED STATES

## Abstract

Influenza is associated with severe illness, death, and economic burden. Sentinel surveillance systems have a central role in the community since they support public health interventions. This study aimed to describe and compare the influenza-coded primary care consultations with the reference index of influenza activity used in Portugal, General Practitioners Sentinel Network, from 2012 to 2017. An ecological time-series study was conducted using weekly R80-coded primary care consultations (according to the International Classification of Primary Care-2), weekly influenza-like illness (ILI) incidence rates from the General Practitioners Sentinel Network and Goldstein Index (GI). Good accordance between these three indicators was observed in the characterization of influenza activity regarding to start and length of the epidemic period, intensity of influenza activity, and influenza peak. A high correlation (>0.75) was obtained between weekly ILI incidence rates and weekly number of R80-coded primary care consultations during all five studied seasons. In 3 out of 5 seasons this correlation increased when weekly ILI incidence rates were multiplied for the percentage of influenza positive cases. A cross-correlation between weekly ILI incidence rates and the weekly number of R80-coded primary care consultations revealed that there was no lag between the rate curves of influenza incidence and the number of consultations in the 2012/13 and 2013/14 seasons. In the last three seasons, the weekly influenza incidence rates detected the influenza epidemic peak for about a week earlier. In the last season, the GI anticipated the detection of influenza peak for about a two-week period. Sentinel networks are fundamental elements in influenza surveillance that integrate clinical and virological data but often lack representativeness and are not able to provide regional and age groups estimates. Given the good correlation between weekly ILI incidence rate and weekly number of R80 consultations, primary care consultation coding system may be used to complement influenza surveillance data, namely, to monitor regional influenza activity. In the future, it would be interesting to analyse concurrent implementation of both surveillance systems with the integration of all available information.

## Introduction

Influenza is a serious public health problem associated with severe illness and death in high-risk populations. The impact also extends to the economic sphere as a result of the decrease in labour productivity and an overload on health and social care services [1–4]. Early detection of epidemics is key to relieving this burden and allows for timely implementation of prevention efforts and control measures to mitigate these effects [4–6].

Sentinel surveillance systems for medical practices are recognized to be the most efficient way to perform influenza surveillance [7]. Sentinel surveillance aims to promptly identify and monitor influenza activity in the community, presenting a central role in support of public health interventions [5,8,9].

Since 1989 to present, the Portuguese General Practitioners Sentinel Network (*Rede Médicos Sentinela*) has provided weekly influenza-like illness (ILI) rate estimates. This network is part of the National Influenza Surveillance System, which integrates epidemiological and virological components [10].

The information produced by the Portuguese General Practitioners Sentinel Network is based on the activity of a sample of National Health System General Practitioners, allows reference weekly and annual incidence rates of several diseases (communicable and non communicable diseases) to be estimated and monitoring of trends and early detection of outbreaks and epidemics within the community to be conducted [10,11]. In addition to national epidemiological surveillance, it constitutes a national database for research into primary care using high quality data collected by volunteers and strongly motivated General Practitioners [11].

The annual population under observation on the Portuguese General Practitioners Sentinel Network and the number of General Practitioners that voluntarily participate in the Sentinel Network is shown in Table 1. Although Sentinel General Practitioners are nationally distributed no geographical representativeness was achieved, but age and sex distribution of the population under observation in the Sentinel Network is similar to the Portuguese population.

**Table 1:** Annual population under observation and number of General Practitioners enrolled in the Portuguese General Practitioners Sentinel Network, 2012‒2016.

	2012	2013	2014	2015	2016
Number of General Practitioners	161	112	133	124	111
Population under observation	270,550	201,461	220,778	201,780	209,807

Recognizing possible bias in surveillance systems is an important step for improving them [12,13], and in order to achieve more accurate surveillance systems, it is always relevant to compare different data sources. In Portugal, an alternative data source of influenza incidences is the primary care consultations coding system, which has been mandatory since 2001 with the implementation of electronic medical records software in the Portuguese National Health System [14]. This electronic clinical records software uses a clinical note format known as SOAP, which consists of four categories: 1.) S-Subjective; 2.) O-Objective; 3.) A-Assessment; and 4.) P-Plan [15,16], and general practitioners use the A-Assessment field to record the problems or diagnoses from each consultation.

On the other hand, from the Portuguese Primary Healthcare Reform conducted after 2005 with the implementation of a pay per performance system, the codification of the consultation with the International Classification of Primary Care (ICPC-2) began to gradually be used in a generalized manner [17–19].

The ICPC-2 is a coding system proposed by the World Organization of Family Doctors classification group for the clinical practice of family physicians. First published in 1987, it was innovative in allowing us to classify reasons for consultation, diagnoses or health problems, and care procedures [20]. Its chapters are based on anatomical systems over aetiology, and it is based on two axes and allows classification with one-letter and two-digit codes [21].

According to ICPC-2, code R80 corresponds to the influenza diagnosis [19,20]. In this context, the analysis of the number of R80-coded primary care consultations can be an alternative source of data of the incidence of influenza for specific population groups or when ILI incidence rates are not available.

This study aimed to describe and compare the evolution of influenza-coded primary care consultations with the reference index of influenza activity used in Portugal, obtained by the General Practitioners Sentinel Network, from 2012 to 2017.

## Materials and methods

An ecological time-series study was conducted using the definitive weekly ILI incidence rate from the General Practitioners Sentinel Network and weekly primary care consultations coded as R80 (ICPC-2) from the 2012/13 to 2016/2017 seasons. ILI incidence rates are expressed per 100,000 inhabitants, however, primary care consultations are presented in absolute numbers since there is not an available denominator to calculate rates.

### General Practitioners Sentinel Network

#### Identification of cases

All ILI cases daily observed by the Sentinel General Practitioner, from their patient list, were notified to the National Influenza Surveillance System. These cases were selected using the best clinical sense and subsequently were centrally validated according to the European Centre for Disease Prevention and Control definition criteria: 1.) sudden onset of symptoms and 2.) at least one of the following four systemic symptoms (fever or feverishness, malaise, headache, and/or myalgia); and 3.) at least one of the following three respiratory symptoms (cough, sore throat, and/or shortness of breath) [22]. Nasopharyngeal swabs were collected to a sample of ILI cases selected by the Sentinel General Practitioner and sent to the National Reference Laboratory for Influenza. No case definition was used for case selection for swabbing.

#### Influenza-like illness incidence rate estimates and Goldstein Index

Along the influenza season, a provisional weekly ILI incidence rate was estimate each week. ILI cases were time allocated accordingly to the onset symptoms date, thus all cases with symptomatic onset date at week (x) and reported until the Thursday of following week (x+1) were counted. The population under observation resulted from the sum of the patient list of each Sentinel General Practitioner who reported a case in week x or indicated no cases to report that week. This incidence rate was used to monitor influenza transmission along the season.

Incidence rates were estimated for Portugal (mainland and islands), and given the low number of cases reported each week, it was not possible to disaggregate them by age group or health region.

After the end of influenza season, weekly ILI incidence rate estimates were recalculated considering all cases with onset date of symptoms referring to week x and reported until the end of influenza season. In this case, the population under observation consisted of the sum of the lists of Sentinel General Practitioners who reported all cases (or indicated they had no cases to report) until the end of the season.

To have a proxy of the weekly influenza incidence rate, Goldstein Index (GI) [23] was also calculated. The GI corresponds to definitive weekly ILI incidence rates multiplied by the percentage of positive influenza cases detected each week on the General Practitioners Sentinel Network.

For this study, the definitive weekly ILI incidence rate from week 40/2012 to 20/2017 week and the GI were used (data updated on 7^th^ July 2017).

### Primary care consultations

#### Identification of cases

Cases were selected through the codification of primary care consultations (Portugal mainland) when “A-Assessment” field was codified as influenza (R80, ICPC-2) [19,20]. These cases were selected using the best clinical sense regarding the ICPC-2 definition criteria: myalgia and cough with no other abnormal physical signs than inflammation of the nasal mucosa and throat membrane and, in addition, three or more of the following: 1) sudden onset (12 hours or less); 2) tremors; 3) chills or fever; 4) prostration and weakness; 5) contacts next with influenza or influenza epidemic; 6) viral culture or serological evidence of influenza virus infection.[19]

### Number of weekly influenza consultations

The weekly provisional number of primary care consultations coded as R80, performed at week x, resulted from the sum of all consultations coded as R80 until Thursday of the following week (x + 1). Cases were allocated to each week accordingly the consultation date.

The number of consultations performed each week was updated continuously. For this study, the weekly number of R80 consultations conducted from week 40/2012 to 20/2017 week (data updated on 12^nd^ July 2017) was used. These data were provided by *Serviços Partilhados do Ministério da Saúde* and are available during influenza season in *Saúde Sazonal* Site (http://saudesazonal.min-saude.pt).

### Statistical analysis

The epidemic and intensity threshold for each indicator were calculated through the Moving Epidemic Method, which recommends using between a minimum of five and a maximum of ten influenza seasons for the epidemic threshold calculation [24,25]. Intensity levels were defined as *low*, *medium*, *high*, and *very high* using the cut-off points agreed on by the European Region [25].

The epidemic and the intensity thresholds were calculated for definitive weekly ILI incidence rate, weekly number of R80 consultations and weekly GI using data of the last five seasons (2012/13 to 2016/17) as data from the R80 consultations was available only for this period of time.

For each influenza season definitive weekly ILI incidence rate, weekly number of R80 consultations and weekly GI were compared to the epidemic and intensity thresholds set up for each indicator. Thus allowed us to identify the start and end of the influenza epidemic, the epidemic peak and the epidemic intensity level of each season accordingly these three different indicators.

For each influenza season (2012/13‒2016/17), correlations (Pearson correlation) and cross-correlations were estimated:

- between definitive weekly ILI incidence rate and weekly number of R80-coded primary care consultations.
- between weekly Goldstein Index and weekly number of R80-coded primary care consultations.

The cross-correlation study allows the identification of the *lag* (delay) between two time-series. If a higher cross-correlation value is found on *lag* 0, the values of the first series (ILI incidence rate or GI) are correlated with the values of the second series (R80 consultations) without delay. If the higher cross-correlation value is found to have a negative *lag*, the values of the first series are correlated with the values of the second series with a delay of *lag* weeks, and if the higher cross-correlation value is found to have a positive lag, the values of the first series are correlated with the values of the second series, and the second series precedes the first in *lag* weeks.

All analyses were performed using R[26] and Mem-package (version1.4).

### Ethics statement

This study was carried out in strict accordance to protect patient privacy and anonymity. Data used on the analysis provided from National Influenza Surveillance System (General Practitioners Sentinel Network) and primary care consultations registries. General Practitioners Sentinel Network has individual data but patient identification is not possible once anonymity is always performed previous to case notification. National Institute of Heath’s Ethical Committee approved surveillance activities performed under the General Practitioners Sentinel Network. Concerning to primary care consultations, data used are not individual but aggregated.

## Results

**Fig 1:**
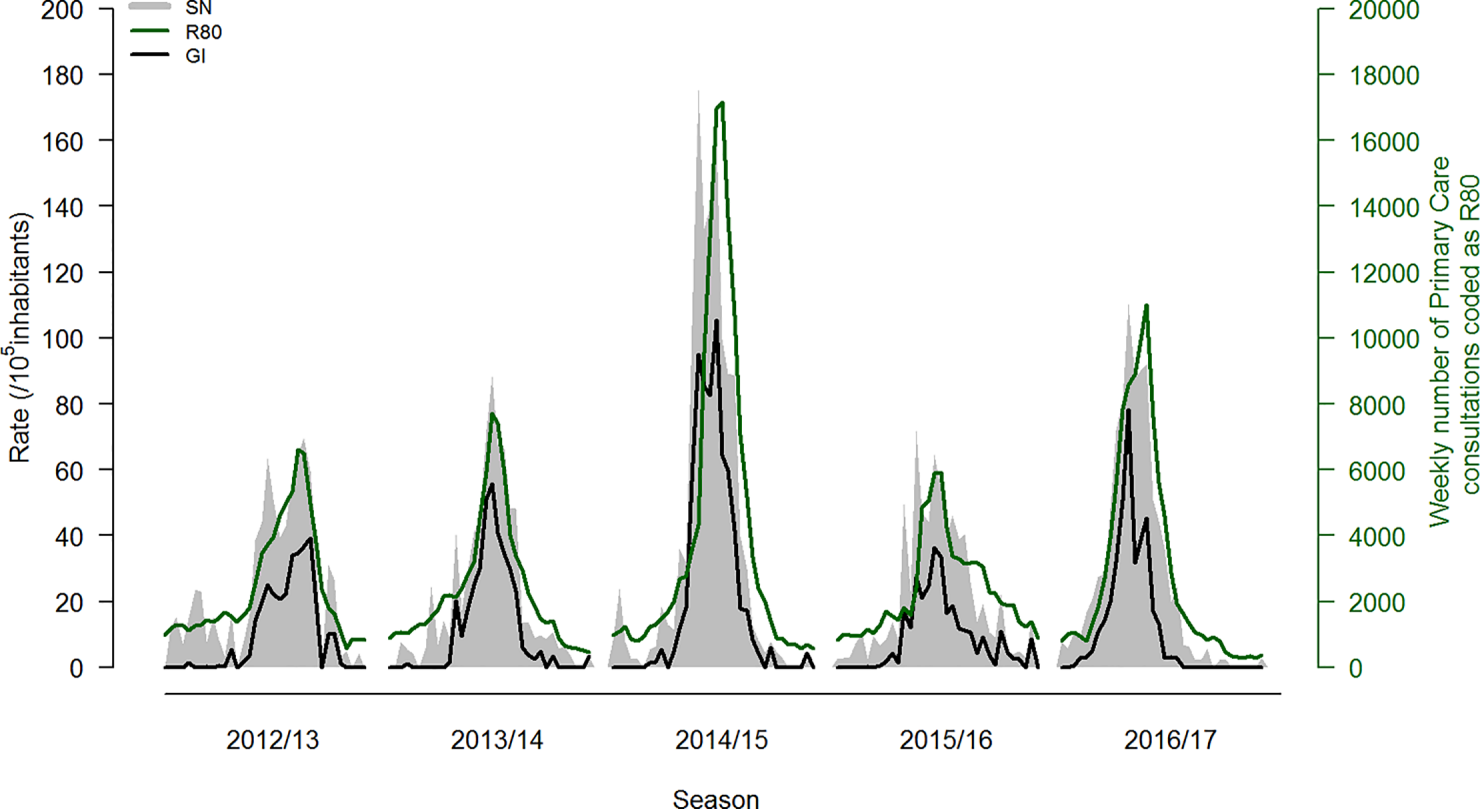
Definitive weekly influenza-like illness (ILI) incidence rate estimated by General Practitioners Sentinel Network (SN), weekly number of R80-coded primary care consultations, and weekly Goldstein Index (GI).

The definitive weekly ILI incidence rates of the General Practitioners Sentinel Network, weekly number of primary care consultations coded as R80, and weekly Goldstein Index for the seasons from 2012/13 to 2016/17 are shown in Fig 1. Characterization of influenza epidemics, occurred between 2012/13 and 2016/17, according with the three different indicators, is summarized in Table 2.

**Table 2:** Characterization of influenza activity from 2012/13 to 2016/17 seasons.

	Weekly number of R80 consultations			Definitive weekly ILI incidence rate			Goldstein Index		
	highest value (week/year)	start and end of epidemic period (week/year)	intensity	highest value /10^5^ (week/year)	start and end of epidemic period (week/year)	intensity	highest value /10^5^ (week/year)	start and end of epidemic period (week/year)	intensity
**2012/13**	6610 (w9/13)	w2/13 to w13/13	medium	69.6 (w10/13)	w1/13 to w12/13	low	39.4 (w11/13)	w2/13 to w12/13	medium
**2013/14**	7708 (w4/14)	w52/13 to w10/14	medium	88.3 (w4/14)	w51/13 to w8/14	medium	55.7 (w4/14)	w51/13 to w8/14	medium
**2014/15**	17153 (w5/15)	w1/15 to w9/15	very high	175.3 (w1/15)	w52/14 to w8/15	very high	105.5 (w4/15)	w2/15 to w9/15	very high
**2015/16**	5934 (w4/16)	w53/15 to w12/16	low	72.0 (w53/15)	w51/15 to w9/16	medium	36.3 (w3/16)	w51/15 to w9/16	low
**2016/17**	10993 (w01/17)	w48/16 to w5/17	medium	110.3 (w51/16)	w46/16 to w4/17	medium	78.3 (w51/16)	w47/16 to w3/17	high

Concordance on intensity levels among the three indicators was achieved in 2 out of 5 seasons. In the 2012/13 season, the R80-coded consultation and the GI overestimated the influenza intensity level when compared with the ILI incidence rate, but in 2015/16 season the opposite was observed (Table 2).

The length of the influenza epidemic was similar between the three indicators; however, when the ILI incidence rate was used, the start of the epidemic was detected at least one week earlier (Table 2).

A high correlation (>0.75) was observed between the ILI incidence rate and the number of R80-coded primary care consultations in all of the studied seasons. In 3 out of 5 seasons, the correlation increased when the ILI incidence rates were multiplied by the percentage of influenza positive cases and the GI was used instead of ILI incidence rate (Table 3).

**Table 3:** Correlation between definitive weekly ILI incidence rate or weekly GI and the weekly number of R80-coded primary care consultations.

	2012/13	2013/14	2014/15	2015/16	2016/17
**Definitive weekly ILI incidence rate vs weekly number of R80 consultations**	0.90	0.95	0.82	0.78	0.95
**Weekly GI vs weekly number of R80 consultations**	0.95	0.94	0.85	0.86	0.86

***Note*:** Pearson´s coefficients were reported.

The cross-correlation values between weekly ILI incidence rate and the number of R80-coded primary care consultations revealed that in the 2012/13 and 2013/14 seasons there was no *lag* between either indicators. In the last three seasons, however, the weekly ILI incidence rate detected influenza epidemic peak for about one week earlier than R80 consultations (0.92; 0.82; 0.96) (S1 Table) and in the last season the influenza peak epidemic detection was for about two weeks earlier when GI was compared to R80 consultations (0.90) (S2 Table).

## Discussion

For the five studied influenza seasons, characterization of influenza activity, regarding to start and length of epidemic, epidemic peak and intensity, was similar when comparing the number of R80-coded primary care consultations and the weekly ILI incidence rate estimated by General Practitioners Sentinel Network or the GI. Similar results were found in another study with good agreement between weekly incidence rates obtained from the diagnoses registered in the primary care electronic medical records and data from Sentinel Network, presenting a correlation coefficient of 0.88 [27].

Even so, in 2012/13, number of R80-coded primary care consultations overestimated influenza intensity (Table 2). Other studies had also observed a trend to overestimate some results of the Sentinel Network in primary care consultations [27,28]. This difference may be explained by the possible greater accuracy in the identification of influenza cases by the General Practitioners Sentinel Network, given their motivation and training [11]. On the other hand, due to the low number of seasons used, we can’t exclude that very high or very low weekly ILI rate values could under or overestimate the baseline threshold [25], which may explain the lack of accordance of epidemic intensity in 2012/13 and 2015/16 seasons between weekly ILI incidence rate and weekly R80 consultations. We highlight that the highest values of ILI incidence rate and R80 consultations registered in both seasons were near to the moderate threshold, which in our point of view, reduce the importance of the observed differences.

Given that in the Portuguese General Practitioners Sentinel Network, case notification and incidence rate estimates are based on the onset of symptoms and not on consultation date, the detection of increased influenza activity above the epidemic threshold was at least one week earlier than that observed with R80. This fact is of major importance as it allows for early control measures implementation and confirms the importance of using the Sentinel Network in influenza surveillance. Among the different health surveillance systems, Sentinel Networks with their practice of frequent reporting and making wise use of low resources, allows timeliness information to be produced and are therefore of great utility in the early detection of an influenza epidemic [10]. Integration of virological and epidemiological data, as is done in the Portuguese General Practitioners Sentinel Network, increases the specificity of the Influenza case definition since the ILI incidence rate is interpreted according the influenza detection. Moreover, viral identification and characterization in each season is referred to in previous literature as the basis for any influenza surveillance system. This basis is needed in order to expedite the challenges of viral changes and need for vaccine updates, variations in the number and severity of people affected, and potential risks of a pandemic [7,29,30].

In a surveillance system, it is imperative to get information as close as possible to that obtained from a random and representative sample of the population [12]. In this context, general practitioners' voluntary participation, in contrast to a random representative selection, has been seen as a challenge for reducing bias in estimates of disease incidence in surveillance networks [13]. Although the age and sex distribution of the population under observation in the Sentinel Network is similar to the Portuguese population no geographical representativeness was achieved. The Portuguese General Practitioners Sentinel Network does not allow ILI incidence rate estimates by age groups and does not cover the entire country. This is probably associated with less representativeness regarding the geographical area since other geographical network-dependent factors point to other Sentinel Networks [31]. The good geographic coverage may be a positive aspect of the Primary Care consultation coding system. The use of universal surveillance systems is not consensual and the World Health Organization recommends countries that use them to select a few healthcare facilities as sentinel sites to gain efficiency and improve the quality of the data [7]. However, universal systems allow a greater population coverage (assuming overlap in the real magnitude of the problem), a more immediate data access (<24 h), and a deeper analysis of information (such as age, sex, comorbidities, or regions), which may contribute to facilitating faster public health decisions [27]. Considering the good correlation between R80 consultations and ILI incidence rate or GI observed during the last five influenza seasons, R80 consultations disaggregated by age groups and regions can be useful, in conjunction with *classical* indicators, in order to monitor influenza epidemic.

## Conclusions

This study seems to indicate that the number of R80-coded primary care consultations can be an alternative source of influenza incidence data. However, when comparing the R80 method with the reference index of influenza activity used in Portugal, General Practitioners Sentinel Network, not all of beneficial points of the latter can be included.

Sentinel Networks are irreplaceable elements in influenza surveillance since they integrate clinical, virological, and epidemiological data but often lack representativeness. In turn, the electronic coding of primary care consultations has provided data with great potential [27,28], thus establishing a situation that makes both systems truly complementary.

In the future, it would be interesting to analyse the implementation of both surveillance systems that are integrated and ideally consisting of on time outputs by region, age, gender, and comorbities.

## Supporting information

S1 Table Cross correlation coefficients between weekly ILI incidence rate and weekly R80 consultations number. (DOCX)

S2 Table Cross correlation coefficients between weekly Goldstein Index and weekly R80 consultations number. (DOCX)
