# Supplementary material for: Comparison between influenza coded primary care consultations and national influenza incidence obtained by the General Practitioners Sentinel Network in Portugal from 2012 to 2017
Source: PLoS One. 2018 Feb 13;13(2):e0192681. doi: 10.1371/journal.pone.0192681 (PMC5811043; doi:10.1371/journal.pone.0192681)
Supplement: S1 Table — (DOCX) [file pone.0192681.s001.docx]

**Supporting information**

**S1 Table.** Cross correlation coefficients between weekly ILI incidence rate and weekly R80 consultations number

| **lag** | **2012/13 season** | **2013/14 season** | **2014/15**  **season** | **2015/16 season** | **2016/17 season** |
| --- | --- | --- | --- | --- | --- |
| -12 | -0,41 | -0,56 | -0,47 | -0,37 | -0,34 |
| -11 | -0,43 | -0,48 | -0,44 | -0,27 | -0,32 |
| -10 | -0,42 | -0,39 | -0,38 | -0,17 | -0,29 |
| -9 | -0,38 | -0,30 | -0,30 | -0,09 | -0,23 |
| -8 | -0,23 | -0,21 | -0,18 | 0,03 | -0,12 |
| -7 | -0,03 | -0,08 | -0,05 | 0,16 | 0,01 |
| -6 | 0,12 | 0,06 | 0,14 | 0,30 | 0,16 |
| -5 | 0,27 | 0,22 | 0,36 | 0,47 | 0,36 |
| -4 | 0,42 | 0,37 | 0,60 | 0,63 | 0,57 |
| -3 | 0,54 | 0,57 | 0,81 | 0,77 | 0,75 |
| -2 | 0,70 | 0,76 | 0,90 | 0,78 | 0,90 |
| -1 | 0,84 | 0,90 | 0,92 | 0,82 | 0,96 |
| 0 | 0,90 | 0,95 | 0,82 | 0,78 | 0,95 |
| 1 | 0,80 | 0,86 | 0,64 | 0,63 | 0,82 |
| 2 | 0,64 | 0,72 | 0,47 | 0,54 | 0,66 |
| 3 | 0,46 | 0,52 | 0,26 | 0,39 | 0,47 |
| 4 | 0,30 | 0,31 | 0,07 | 0,23 | 0,25 |
| 5 | 0,17 | 0,11 | -0,09 | 0,10 | 0,07 |
| 6 | 0,01 | -0,05 | -0,22 | -0,08 | -0,10 |
| 7 | -0,15 | -0,16 | -0,31 | -0,23 | -0,23 |
| 8 | -0,29 | -0,24 | -0,38 | -0,35 | -0,32 |
| 9 | -0,39 | -0,31 | -0,42 | -0,47 | -0,38 |
| 10 | -0,45 | -0,37 | -0,45 | -0,49 | -0,40 |
| 11 | -0,43 | -0,44 | -0,47 | -0,56 | -0,41 |
| 12 | -0,38 | -0,46 | -0,47 | -0,55 | -0,38 |
