# Supplementary material for: Comparison between influenza coded primary care consultations and national influenza incidence obtained by the General Practitioners Sentinel Network in Portugal from 2012 to 2017
Source: PLoS One. 2018 Feb 13;13(2):e0192681. doi: 10.1371/journal.pone.0192681 (PMC5811043; doi:10.1371/journal.pone.0192681)
Supplement: S2 Table — (DOCX) [file pone.0192681.s002.docx]

**Supporting information**

**S2 Table.** Cross correlation coefficients between weekly Goldstein Index and weekly R80 consultations number

| **lag** | **2012/13 season** | **2013/14 season** | **2014/15**  **season** | **2015/16 season** | **2016/17 season** |
| --- | --- | --- | --- | --- | --- |
| -12 | -0,45 | -0,56 | -0,46 | -0,39 | -0,29 |
| -11 | -0,45 | -0,51 | -0,43 | -0,31 | -0,28 |
| -10 | -0,45 | -0,45 | -0,39 | -0,20 | -0,26 |
| -9 | -0,42 | -0,38 | -0,32 | -0,11 | -0,20 |
| -8 | -0,29 | -0,29 | -0,21 | 0,01 | -0,12 |
| -7 | -0,10 | -0,14 | -0,09 | 0,15 | 0,01 |
| -6 | 0,06 | 0,03 | 0,10 | 0,27 | 0,17 |
| -5 | 0,21 | 0,22 | 0,33 | 0,43 | 0,36 |
| -4 | 0,39 | 0,40 | 0,58 | 0,57 | 0,56 |
| -3 | 0,55 | 0,61 | 0,80 | 0,70 | 0,76 |
| -2 | 0,71 | 0,80 | 0,92 | 0,79 | 0,90 |
| -1 | 0,87 | 0,93 | 0,95 | 0,86 | 0,88 |
| 0 | 0,95 | 0,94 | 0,85 | 0,86 | 0,86 |
| 1 | 0,90 | 0,83 | 0,67 | 0,71 | 0,68 |
| 2 | 0,76 | 0,68 | 0,47 | 0,56 | 0,48 |
| 3 | 0,57 | 0,48 | 0,25 | 0,36 | 0,26 |
| 4 | 0,40 | 0,27 | 0,06 | 0,14 | 0,06 |
| 5 | 0,27 | 0,09 | -0,09 | 0,01 | -0,10 |
| 6 | 0,10 | -0,05 | -0,21 | -0,12 | -0,23 |
| 7 | -0,07 | -0,16 | -0,29 | -0,24 | -0,31 |
| 8 | -0,21 | -0,25 | -0,35 | -0,31 | -0,34 |
| 9 | -0,32 | -0,32 | -0,40 | -0,41 | -0,36 |
| 10 | -0,38 | -0,38 | -0,43 | -0,43 | -0,36 |
| 11 | -0,38 | -0,44 | -0,44 | -0,48 | -0,33 |
| 12 | -0,35 | -0,45 | -0,44 | -0,48 | -0,26 |
